# A Comparative Study on the Thermal Energy Storage Performance of Bio-Based and Paraffin-Based PCMs Using DSC Procedures

**DOI:** 10.3390/ma13071705

**Published:** 2020-04-05

**Authors:** Mona Nazari Sam, Antonio Caggiano, Christoph Mankel, Eddie Koenders

**Affiliations:** 1 Institut für Werkstoffe im Bauwesen, Technische Universität Darmstadt, 64287 Darmstadt, Germany; sam@wib.tu-darmstadt.de (M.N.S.); mankel@wib.tu-darmstadt.de (C.M.); koenders@wib.tu-darmstadt.de (E.K.); 2 CONICET, LMNI, INTECIN, Facultad de Ingeniería, Universidad de Buenos Aires, Ciudad Autónoma de Buenos Aires C1127AAR, Argentina

**Keywords:** thermal-energy storage (TES), phase change materials (PCMs), latent enthalpy, melting temperature, differential scanning calorimetry, IEA method

## Abstract

Thermal-Energy Storage (TES) properties of organic phase change materials have been experimentally investigated and reported in this paper. Three paraffin-based Phase Change Materials (PCMs) and one bio-based PCM are considered with melting temperatures of 24 °C, 25 °C and 26 °C. Sensible heat storage capacities, melting characteristics and latent heat enthalpies of the studied PCMs are investigated through Differential Scanning Calorimetry (DSC) measurements. Two alternative methods, namely the classical dynamic DSC and a stepwise approach, are performed and compared with the aim to eliminate and/or overcome possible measurement errors. In particular, for DSC measurements this could be related to the size of the samples and its representativity, heating rate effects and low thermal conductivity of the PCMs, which may affect the results and possibly cause a loss of objectivity of the measurements. Based on results achieved from this study, clear information can be figured out on how to conduct and characterize paraffin and bio-based PCMs, and how to apply them in TES calculations for building applications and/or simulations. It is observed that both paraffinic and bio-based PCMs possess a comparable TES capacity within the selected phase transition temperature, being representative for the human thermal comfort zone. The phase change of bio-based PCMs occurred over a much narrower temperature range when compared to the wider windows characterizing the paraffin-based materials. Bio-based PCMs turned out to be very suitable for building applications and can be an environmentally friendly substitute for petroleum-based PCMs.

## 1. Introduction

The global challenge to strongly cut back the use of fossil fuels with the aim to implement renewable resources and to neutralize greenhouse gas emissions make energy efficiency a key issue that is at the center of our society [1,2]. According to the EU commission, heating and cooling the residential and non-residential sector accounts for half of the EU’s energy consumption, while about 84% of it is still generated from fossil resources [3]. Since the introduction of the new EU Buildings Directive 2019/2021 [4], all member states are obliged to guarantee that all new constructions are designed as “Nearly Zero Energy Buildings” (NZEBs), from the beginning of 2021. This obligation has been already applied to non-residential buildings from the beginning of 2019. Therefore, the development of new and smart energy storage solutions and technologies, with the aim to use environmental thermal energy more efficiently, and to balance out daily heating/cooling demands, are worth investigation for building applications [5,6].

One promising technique available for Thermal Energy Storage (TES) applications is by implementing Phase Change Materials (PCMs) in construction and building composites [7]. PCMs can be used to store large quantities of heat, not only through their sensible capacity, but also (sometimes predominantly) via their latent storage property [8]. As PCMs are capable of storing large amounts of latent heat at constant temperature, they are contributing to the energy efficiency and thermal comfort of residential and non-residential buildings, by balancing out daily environmental heat demands [9]. Consequently, the passive storage/release of latent heat through phase transitions from solid to liquid or vice versa, allows to save considerable amount of primary energy [10]. The principle of latent TES can be employed in a wide range of applications [11], such as, solar heating systems [12], building air conditioning [13], building envelope [14], production of energy-saving cementitious composites [15] and high porous insulation systems [16], waste heat recovery in residential and non-residential sectors [17], and many other applications [18].

Designing large-scale practical applications for passive latent thermal energy storage systems requires in-depth knowledge on the thermal characteristics of PCM before, during and after its phase change. Therefore, an accurate and correct determination of the thermal properties of PCM systems is crucial to efficiently design composite systems or devices that use latent TES [19].

Differential Scanning Calorimetry (DSC) is an effective method to characterize the thermal behavior of PCMs, and to determine their TES capacities, in terms of transition temperature, enthalpy and specific heat, and its stability throughout the various melting and crystallization cycles. The heating and cooling rates of DSC measurements are typically much faster than in real applications, while the sample mass is also very small (less than 90 mg), which might lack representativity for real size applications [20,21]. Some commercially calorimeters are already available on the market for measuring and analyzing larger sample masses with a very high precision and adopting quite low heating rates (as low as 0.01 K/min). Actually, samples with a large mass implicitly show a lower error ratio resulting from reduced sample inhomogeneity. However, larger samples may also strongly affect the course of the measured phase change temperatures (i.e., the onset temperature or the maximum one). It follows that DSC measurements can be affected by changing the heating/cooling rate and/or sample mass. They mainly influence the thermal equilibrium status in the sample, thus producing a shifting of the phase change temperature and also lead to non-realistic shapes of the heat capacity and/or enthalpy temperature responses *h(T)* [22]. Moreover, DSC test results depend on further factors such as sample preparation, correct calibration of the experimental set-up, and many other factors that should, in fact, be standardized in order to achieve comparable and objective results, under different test environments [23]. Therefore, developing an appropriate and objective methodology, in this field, is essential to improve the accuracy of the PCM characterization procedure and to make measurement errors negligible [24].

An alternative method that overcomes the aforementioned DSC limitations (i.e., mass influence and heating rate effects) is the T-History Method (THM), which was originally developed by Yinping and Yi [25]. The method records the time–temperature evolutions of PCM samples against a well-known reference material, usually water. THM easily allows to evaluate the heat capacity, temperature of melting/crystallization, enthalpy and phase change temperature. The accuracy and soundness of this method was evaluated by many researchers, see for example [26,27,28]. Hong et al. [29] verified the accuracy of a modified THM for several PCMs, having different freezing patterns. A further improvement of the THM measurement technique was developed by Stankovic and Kyriacou [30]. A procedure to numerically correct the enthalpy-temperature response of PCMs obtained from THM was developed by Tan et al. [31]. Then, a critical comparison between DSC and THM was done by Rathbeger et al. [32]. The authors mainly concluded that the key difference between both methods is represented by the sample size of the investigated material, and the temperature profile to which they are subjected. THM samples are much larger than DSC samples (about 1000 times), while also constant heating and/or cooling temperatures are applied. In addition, THM minimizes heating/cooling rate effects, overcomes sample size issues and can be used for low thermal conductivity samples. The only drawback of THM, which is actually crucial for standardized measurements and test procedures, is that the accuracy of the lab results largely depends on the measuring procedures and the self-built calorimeters. For this reason, measurements from different laboratories can differ a lot and are generally not comparable [33].

The main objective of this work is to investigate the thermo-physical properties and heat storage capacity of a representative organic bio-based (non-paraffin) PCM, in terms of phase-change enthalpy, specific heat capacity and melting temperature, using different experimental setups. The influence of various parameters, involved in DSC measurements, are compared with results done on three comparable paraffin waxes. The measurements are carried out in accordance with the currently available standard of the International Energy Agency (IEA), i.e., under the Task 42 Annex 29, to characterize the thermal-energy properties of the PCMs under discussion. Heat-flux DSC dynamic measurements and DSC stepwise procedures are considered and compared to scrutinize the aforementioned aims.

To the Authors’ best knowledge, only a few studies on thermal analysis (even under the still not published final version of the IEA-SHC 42 Annex 29 Standard for common PCMs) are so far available in scientific literature dealing with the use of bio-based PCM. Moreover, no Standard is currently available on the thermal analysis of organic PCMs, but there are some discussions going on in this direction. In this context, with the current paper, the authors like to contribute to this discussion by presenting the differences in DSC responses between three commonly used petroleum-based PCMs (paraffinic) and one novel bio-based PCM. Especially highlighting the differences in TES characteristics and data of various kinds of PCMs, in a general sense. Thus, this document tried to examine if the currently employed procedures can also be applied to other types of PCMs, such as the considered bio-based one investigated in this work. In addition, the presented results lead to demonstrate the feasibility of completely substituting petroleum-based PCMs with a more environmentally friendly bio-based one. This aspect may open doors for a new scenario in research for developing novel PCMs, that can be employed in thermal storage materials for building construction materials, which can be more sustainable, eco-friendly and energy-saving.

The paper is structured as follows: Section 2 reports Materials and Methods of the experimental program and outlines the key thermophysical characteristics of the investigated materials and shows the overall experimental program. Then, in Section 3, the experimental results for both paraffin and bio-based PCMs are described following the dynamic DSC procedure. Section 4 outlines the results of the stepwise approach where some comparisons between the two alterative procedures are conducted to characterize the TES of the PCMs. Finally, concluding remarks and future developments of this research are addressed in Section 5.

## 2. Materials and Methods

In this section, the employed materials, methods and experimental program, considered for analyzing the TES properties of the selected PCMs, are presented. Particularly, two types of organic phase change materials are analyzed, namely three Paraffin Waxes and one Bio-based PCM, which have physical, kinetic, chemical and economic relevance for constructions and building application in civil engineering [34,35].

### 2.1. Paraffin Wax

Three commercial paraffin-based waxes (RT-series^®^ by Rubitherm GmbH, Berlin, Germany) are used as reference PCMs. These commercial products, having a melting temperature *T_m_* that ranges between 22 °C and 26 °C (suitable for enhancing the thermal comfort in building applications) are selected for this study. More specifically, RT24, RT25 and RT26 are taken into consideration which are characterized with a *T_m_* of 24 °C, 25 °C and 26 °C, respectively.

### 2.2. Bio-based PCM

One bio-based PCM has also been investigated in this work as an eco-friendly alternative to the petroleum-based paraffin wax. The bio-based material, viz. PureTemp25 by PureTemp LLC (Minneapolis, MN, USA), made of natural oils with a *T_m_* of 25 °C, is used, while its results can be directly compared to RT25. The thermal properties of the RTs and the bio-based PCM are listed in Table 1.

### 2.3. Methods

**Figure 1:**
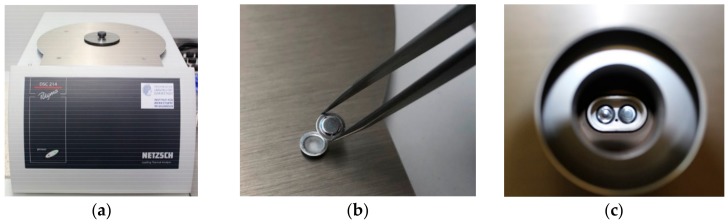
(**a**) Differential Scanning Calorimetry (DSC) 214 Polyma equipment from company Netzsch (Selb, Germany, with a T work range −170 °C to 600 °C — Heating/Cooling rate 0.001 K/min to 500 K/min — Indium Response Ratio > 100 mW/K — Resolution (technical) 0.1 µW — Enthalpy precision ±0.1% for indium, ± 0.05% to ±0.2% for most samples), (**b**) aluminum sample holders (maximum volume capacity of 40 µL) and (**c**) position into the DSC device.

This section reports the methods used to investigate the TES properties of the PCMs presented in Section 2.1 and Section 2.2. Two alterative heat-flux DSC methods (Figure 1), namely the dynamic and a stepwise method, have been considered for this purpose. The German Standards DIN 51005 [38] and DIN 51007 [39] have been considered as a reference to perform the DSC tests (generally applied for this test method to any kind of material), while, the IEA standard procedure [40] is followed to determine the heat storage capacity of PCMs.

**Figure 2:**
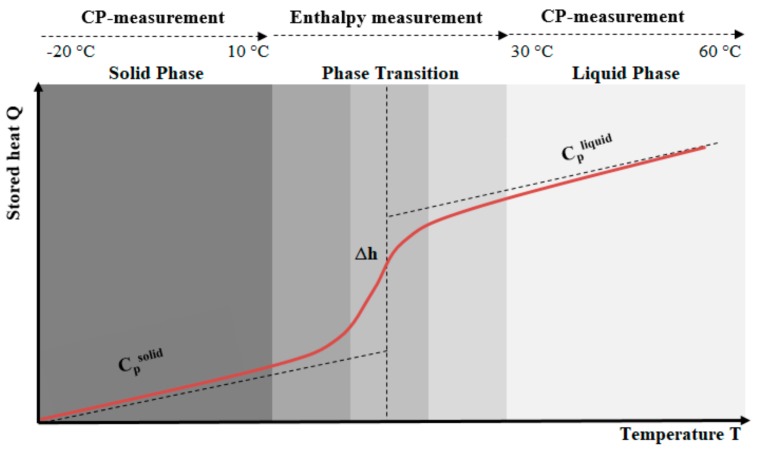
Separation of the measurements in 3 phases: (i) heat (sensible) capacity in solid phase, (ii) heat (latent) capacity under phase change, and (iii) heat (sensible) capacity in liquid phase according to DIN EN ISO 11357-3 [41].

Characterizing the PCMs was done by measuring three cycles over three different temperature ranges: (i) solid phase (−20 to 10 °C); (ii) phase change/transition (melting and/or crystallization), (10 to 30 °C); and (iii) liquid phase (30–60 °C). Figure 2 shows these three ranges that occur during the DSC heating/cooling tests, and also describes the typical enthalpy and specific heat capacity evolutions of non-isothermal PCMs.

It may be worth highlighting that only for the dynamic DSC tests, and following the IEA indications [40], each sample measurement consisted of 3 (DSC) cycles over the pre-defined temperature range (−20 to 60 °C). Particularly, the first cycle was performed to eliminate previous thermal histories of the specimen; the second cycle was carried out to identify the TES characteristics; and finally, the third one was done mainly to check the reproducibility of the results and the possible appearance of cyclic chemical instability of the material.

#### 2.3.1. Dynamic DSC Method and Evaluation of TES Parameters

The most common way to operate DSC tests is by performing the experimental procedure with a constant heating/cooling rate. This is known as “dynamic DSC”, since the heat transfer (energy) is evolving without a necessary thermodynamic equilibrium inside the analyzed sample. However, DSC measurements with a fast and dynamic process and occurrence of phase change phenomena can be affected by this lack of thermodynamic equilibrium, and, hence can provide non-realistic enthalpy temperature data *h (T).* The shape of the latter can be largely affected while the melting point may be systematically shifted towards higher values for the heating cycles, or lower crystallization temperatures for the cooling process.

This will result in significant errors in the temperature-dependent measurements, such that the heat supply/release, referred to each temperature record, cannot be objectively attributed to the real TES values. For this reason, carrying out measurements with different heating rates and mass variations can provide good information about the influence of the measured variables, detects the needed thermal equilibrium status of the material and verifies the soundness of the measured data.

From a practical point of view and to avoid the above-mentioned complications, the heating (or cooling) rate must be low enough to allow the sample to be measured close to the various isothermal states and with reasonable accuracy. The procedure described in the IEA standard [40] gives some direction in this sense and it was used to control/solve heat rate issues. This was done by changing the heating rate slowly from 10.0 K/min (high rate) to 0.125 K/min (low rate), or even lower, until the temperature difference between two inflection points of consecutive enthalpy curves (i.e., the peaks of the corresponding *c_p_-T* curves), is lower than a predefined threshold (normally fixed by the standard to 0.2 K). Thus, the maximum permissible heating rate will be the minimum heating rate of two consecutive heating curves which comply with the above criterion [40]. A heating rate of 10 K/min was adopted for the measurements in the sensible range following the DIN 51007 [39]. In these measurement conditions, large quantity samples were tested in order to achieve a proper signal from the device.

The enthalpy change (at both sensible and latent stage), *dH(T),* of a sample, can be evaluated by integrating the heat flow registered during the DSC measurements. It can be assumed that a small variation of enthalpy *dH* (or the specific one, *dh*) is equal to a small amount of heat *dQ* (or the specific one, *dq*) added/released [42,43]. This is valid for those processes characterized at constant pressure, as it happens during DSC experiments.

Therefore, it can be written that:

(1) $$dH=dQ\;\;\;\;m^{sp}dh=m^{sp}dq$$

being

(2) $$dq=c_{p}^{sp}\cdot dT$$

where *m^sp^* is the sample mass, chosen as small as possible to comply with the instrument-specific requirements and to reach the state of equilibrium more quickly [39]. However, *m^sp^* should be large enough to guarantee the representativity of the material sample. Furthermore, *dT* represents a small variation of temperature, and $c_{p}^{sp}\left(T\right)$ the specific heat capacity.

Based on the recorded heat flow (DSC signal), the specific heat capacity of the sample $c_{p}^{sp}\left(T\right)$ can be easily determined from Equation (2), as follows

(3) $$c_{p}^{sp}=\frac{dq}{dT}\underset{\mathrm{under}\;\mathrm{finite}\;\Delta T}{\to }c_{p}^{sp}=\frac{\Delta q}{\Delta T}$$

while the enthalpy variations have been directly evaluated from Equation (1).

In general, changes in enthalpy (latent heat) or the specific heat capacity (sensible heat) of an examined sample are determined by recording the absorbed heat between two equilibrium states, assigned as baselines of the acquired measurement curves. It is worth highlighting that the baseline-construction due to the specific heat capacity, measurements outside its melting range, is determined by performing three measurements: (1) empty measurement, (2) calibration measurement, and (3) real sample measurement for each temperature range (more details are available in [39]). This is due to the correction of measurement results possible affected by asymmetries and to compensate device specific errors.

#### 2.3.2. Stepwise DSC Method and Evaluation of TES Parameters

**Figure 3:**
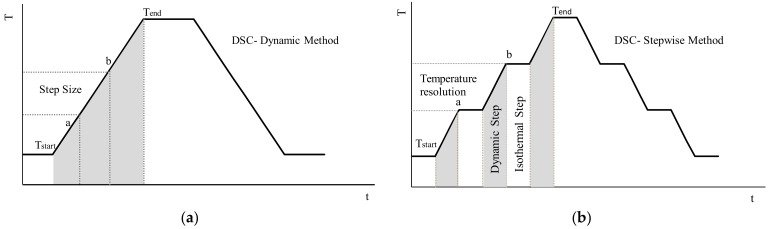
*T(t)* program for (**a**) dynamic DSC method and (**b**) stepwise DSC method.

A stepwise method is used as an alternative for reducing the heating rate effect that is affecting the measurements of dynamic DSC tests. In the stepwise procedure, the net heat applied in a certain temperature interval, is the same as the amount considered in the dynamic DSC tests (see Figure 3a,b). However, the whole interval is sub-divided into small sub-steps. This will favor the accumulation time to reach the thermodynamic equilibrium, between each temperature step, and will improve the temperature resolution (indicated in Figure 3b), which was assumed to be 1 K in this study [44]. In this procedure, isothermal (equilibrium) states are always awaited between two subsequent temperature steps (i.e., 1 K). The waiting needed to reach this state of equilibrium mainly depends on the selected (temperature) step and can only be determined empirically. In this work, the waiting times were taken 10 min at the beginning of the melting and 20 min by reaching the melting peaks. More details are described in Section 4.

The total specific heat, supplied over the individual intervals, is obtained by summarizing the heat increments supplied at each sub-step $\Delta q^{\mathrm{stepwise}}$. Thus, the experimental approach used in the stepwise method to analyze the *h(T)* response of the materials, along with the melting peak temperature *T_m_*, follows the same relationships as proposed for the dynamic DSC test:

(4) $$\Delta H=m^{sp}\Delta h=m^{sp}\Delta q^{\mathrm{stepwise}}$$

### 2.4. Experimental Program

**Figure 4:**
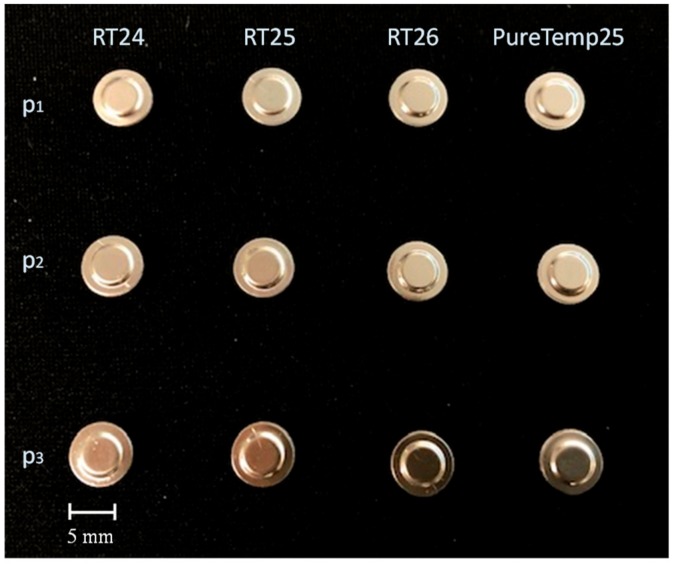
DSC specimens for the measurement of specific heat capacity and enthalpy of Phase Change Materials (PCMs) following the dynamic and stepwise procedures.

For the measurement of each parameter pair (*c_p_*, *h*) three samples per material (paraffin-based RT24, RT25, RT26 and bio-based PureTemp25, as shown in Figure 4) were taken into account. Table 2 reports an overview of the full experimental program considered for the dynamic and stepwise method, respectively. The table gives information regarding the considered heating/cooling rates (in both dynamic and stepwise methods), which range from 10 K/min to 5 K/min, 2 K/min, 1 K/min, 0.5 K/min, 0.25 K/min, and 0.125 K/min. Furthermore, the investigated parameters, considered masses and test type of DSC method are also highlighted in the table.

## 3. Dynamic DSC Results

### 3.1. Heating Rate Effect

Dynamic DSC tests were performed for all PCMs (3 paraffin-based and 1 bio-based), considering several heating rates (ranging from 10 K/min to 0.125 K/min) and various masses. The effect of the heating rate, between 10 °C to 50 °C, was investigated for only one of the samples per each PCM type (labelled as P1 in Table 2 of either RT24, -25, -26 or PureTemp 25).

**Figure 5:**
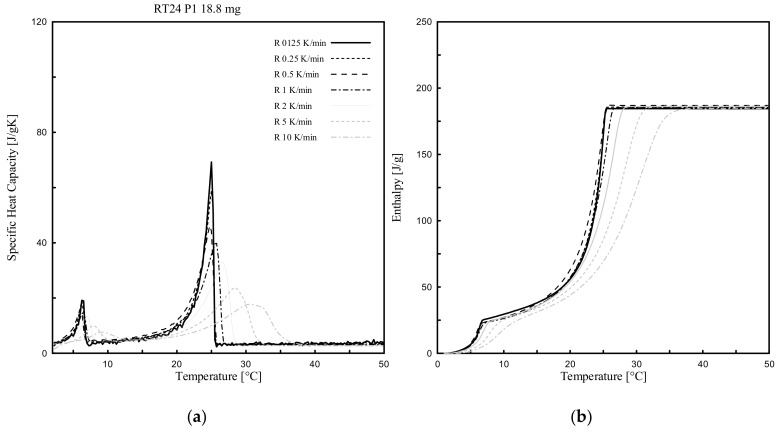
Dynamic DSC measurements: (**a**) *c_p_(T)* and (**b**) *h(T)* curves (latent only) with different heating rates for RT24.

**Figure 6:**
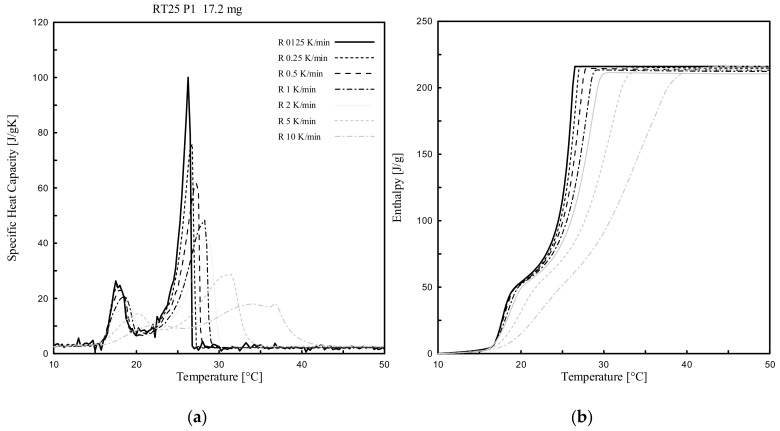
Dynamic DSC measurements: (**a**) *c_p_(T)* and (**b**) *h(T)* curves (latent only) with different heating rates for RT25.

**Figure 7:**
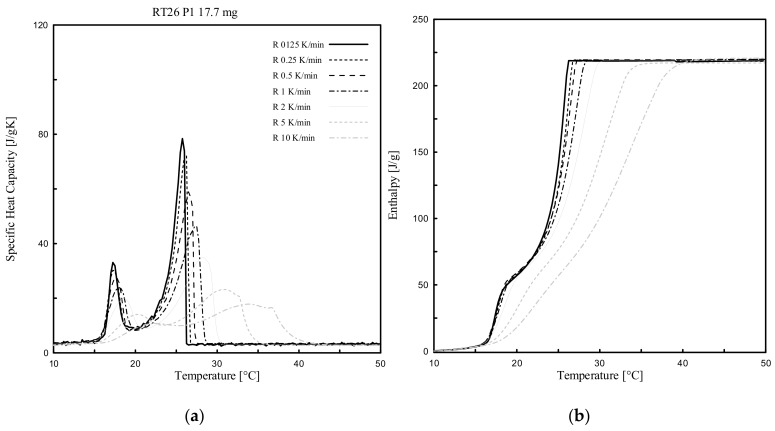
Dynamic DSC measurements: (**a**) *c_p_(T)* and (**b**) *h(T)* curves (latent only) with different heating rates for RT26.

**Figure 8:**
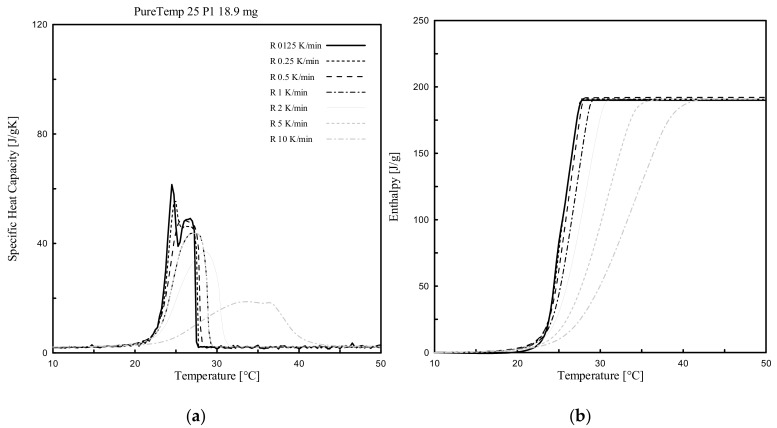
Dynamic DSC measurements: (**a**) *c_p_(T)* and (**b**) *h(T)* curves (latent only) with different heating rates for PureTemp25.

Figure 5, Figure 6, Figure 7 and Figure 8 show the resulting *c_p_(T)* and *h(T)* (latent-only) curves of the samples abovementioned. It can be observed that the melting temperature *T_m_*, defined as the temperature at which the maximum peak of the *c_p_(T)* curve is registered, of all four samples mainly depends on the heating rate. *T_m_* is shifting progressively to lower values with decreasing heating rates. When comparing the different DSC measurements of each sample, the influence of the heating rate on the curves becomes clearly visible. It may be worth mentioning that even the *c_p_(T)* and *h(T)* (latent-only) curves are largely influenced by the rate of heating during the melting transition, the related measured total specific latent heat (enthalpy) is independent on the heating rate.

From the experimental results (Figure 5, Figure 6, Figure 7 and Figure 8) it can be also observed that the acceptable heating rate to be selected in order to stay below the defined threshold value of 0.2 K (i.e., the difference between a high and successive smaller heating rate, as proposed by the IEA standard [40] and described in Section 2.3.1), must be 0.125 K/min (or lower) for RT25 and PureTemp 25, and 0.250 K/min (or lower) for RT24 and RT26. This would reduce as much as possible the heating rate issues for the considered PCMs.

The *c_p_* curves of RT24, -25 and -26 with different heating rates contain mainly two (local) peaks, at different temperatures. These double-peak curves actually become more pronounced for lower heating rates. The first local one, as for each curve and considering 0.125 K/min, was registered at 6.2 °C for RT24 (Figure 5), at 17.6 °C for RT25 (Figure 6) and 17.4 °C for RT26 (Figure 7). The second local (and also maximum) peak, which is representing the melting of paraffin, while adopting a heating rate of 0.125 K/min, was achieved at 25.0 °C for RT24 (Figure 5), at 26.5 °C for RT25 (Figure 6) and 26.2 °C for RT26 (Figure 7).

Similar behavior can be observed from the *c_p_-* and *h-T* (latent-only) DSC curves of the bio-based PureTemp25. From the DSC tests, two adjacent peaks appear in the *c_p_-T* curves. The first melting temperature of the highest peak was measured at 24.6 °C (Figure 8), while the second local (not maximum) peak was registered at a higher temperature of 26.2 °C. The actual melting peak temperature of 24.6 °C was close to the declared value in the datasheet, which is 25 °C (Table 1).

The presence of a local (not maximum) peak, that was detected for both PCM types, could be the result of either a mixture of different compounds, having different chain lengths, or a possible occurrences of solid–solid latent phase transitions prior the melting.

These results also show that the declared melting temperatures in the datasheets (Table 1) are lower than the ones measured with the dynamic DSC tests. This was expected, since measuring the exact melting peak temperature requires a heating rate that should be as low as possible, meaning theoretically nearly 0 K/min. In practice, this is not feasible, since extremely low heating rates result in very weak signals and characterized by huge noise on the measured signal and implicitly limits the choice of the lowest possible heating rate to be assigned.

### 3.2. Mass of Sample Effect for RT25 and PureTemp 25

The effect of the sample mass in the dynamic DSC experiments is discussed in this subsection. Paraffin-based RT25 and bio-based PureTemp 25 were considered for this aim and two masses per each PCM were investigated. Tests were performed considering different heating rates (again ranging from 10 K/min to 0.125 K/min), with temperatures raising between 10 °C and 50 °C; 7.9 mg and 16.3 mg samples were tested for RT25, while 10.1 mg and 18.9 mg for the PureTemp 25.

**Figure 9:**
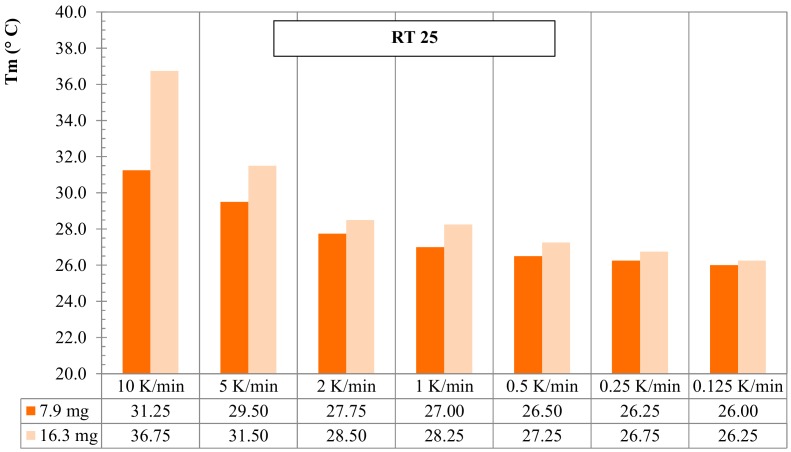
Melting peak temperature *T_m_* for dynamic DSC measurements with several heating rates and 2 different masses for RT25.

Figure 9 shows the comparative evolution of the melting peak temperatures, *T_m_*, against the different heating rates by considering the aforementioned two masses (low mass 7.9 mg and high mass 16.3 mg, respectively). It can be observed that the measured melting points, through the DSC tests for the small sample mass (7.9 mg), rather quickly approximates the melting peak temperature (i.e., 26.0 °C of the lowest heating rate 0.125 K/min). Hence, *T_m_* was 26.50 °C at 0.50 K/min and 26.25 °C at 0.25 K/min. Contrarily, the sample with the higher mass (16.3 mg) showed more influence of the heating rate: i.e., *T_m_* = 27.25 °C at 0.50 K/min, 26.75 °C at 0.25 K/min and 26.25 °C at 0.125 K/min.

**Figure 10:**
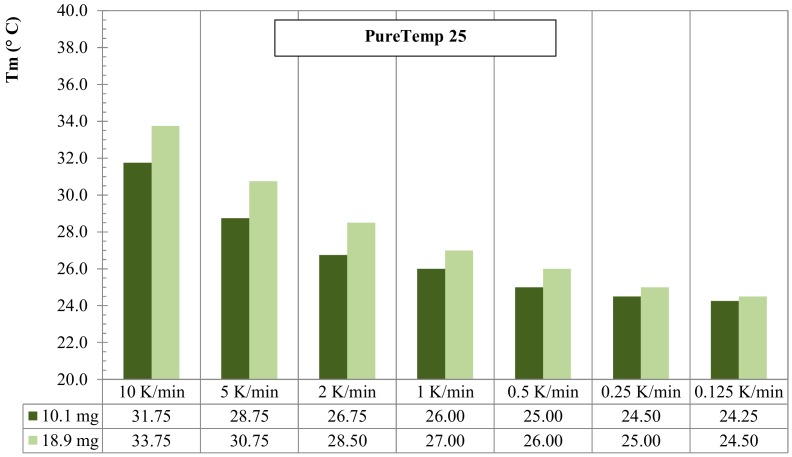
Melting peak temperature *T_m_* of dynamic DSC measurements with several heating rates and 2 different masses for PureTemp25.

Also, for PureTemp 25 the comparison of melting peak temperatures, *T_m_*, against the various heating rates and two different masses (10.1 mg and 18.9 mg, respectively) is presented in Figure 10. As for RT25, the sample with the lower mass (10.1 mg) showed a less influence of the heating rate on the melting peak temperature (e.g., *T_m_* = 24.50 °C at 0.25 K/min and 24.25 °C at 0.125 K/min). For the higher mass (18.9 mg) this was, *T_m_* = 25.00 °C and 24.50 °C, at 0.25 K/min and 0.125 K/min, respectively.

It should be noted that all curves, which were obtained from measurements with heating rates less than 0.125 K/min, had high fluctuations due to signal noises, which make the evaluation of the data and baseline construction difficult to build up. For this reason, a heating rate of 0.125 K/min was considered as the lowest possible choice at which the instrument could still detect signals that were easy to be analyzed. Moreover, 0.125 K/min, for all tests, provides a proper accuracy to fulfill the IEA standard requirements [40], see Section 2.3.1.

The results in this section showed that the selection of the “heating rate” and “sample mass” arises mainly from a compromise between accuracy of the measurements (enthalpy, specific heat capacities and melting peak values) and mitigation of the data due to heating rate effects. Besides the effect of instrument precision and adopted parameters, the measurement accuracy also depends on the representativeness of the sample, which also requires the analysis of larger masses. However, higher mass samples need a strong reduction of the heating rate for achieving a proper thermal equilibrium and valid TES analysis. From the practical point of view, very low heating rates go along with huge signal noises. Contrarily, in low mass samples, thermal equilibrium can be reached more easily with higher heating rates. Experimentally, the most representative mass should be found first, followed by choosing the proper “noiseless” heating rate.

### 3.3. Specific Heat Capacities, Enthalpy and Melting Points for Heating and Cooling

For each type of PCM, six samples were investigated through dynamic DSC tests under both heating and cooling. Three of them were used for *c_p_(T)* characterization, while the remaining three for *h(T).* Before and after the non-isothermal phase change (either melting or crystallization), a heating/cooling rate of 10 K/min was adopted for all tests to minimize noise ratios in the signal [39]. In the latent stage, the considered heating/cooling rates were of 0.250 K/min for RT24 and RT26 and 0.125 K/min for RT25 and PureTemp 25. These assumptions were done following the IEA standard procedure [40] to mitigate the influence of the heating/cooling rate for each compound, as discussed in previous Section 3.1.

**Figure 11:**
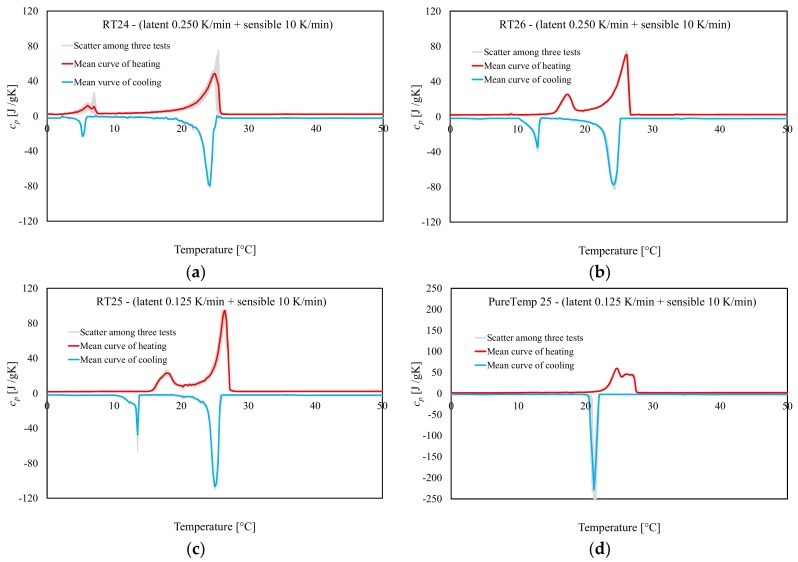
Specific heat capacities following the dynamic DSC tests (for heating between 0 °C and 50 °C and cooling between 50 °C and 0 °C): (**a**) RT24, (**b**) RT26, (**c**) RT25 and (**d**) PureTemp 25. Note: the total specific heat capacities have been constructed by additively linking the sensible parts with the latent ones.

Figure 11 shows the results of dynamic DSC measurements done for the paraffin waxes (RT24, RT25, RT26) and the bio-based PCM (PureTemp 25), for determining the *c_p_* [*J/g × K*] versus *T* [°C] response, in both latent and sensible ranges. All DSC curves are characterized by an almost sensible behavior in the temperature range far from the melting and crystallization points (i.e., in the solid range −20 °C to 10 °C and the liquid range 30 °C to 60 °C), while a non-isothermal latent behavior appears in the phase change region (either with solid-to-liquid and liquid-to-solid responses). It can be also observed that the results show very little (almost negligible) deviation, for all tests, as indicated by the gray area. In the liquid-sensible ranges (i.e., between 30 and 60 °C), the determined specific heat capacities varied between 2.1 and 2.3 J/g × K (for both heating/cooling), for all samples RT24, RT25, RT26 and PureTemp 25. Then, in the lower temperature ranges (solid-sensible responses, between −20 and 10 °C), the *c_p_* values varied between 1.7 and 2.0 J/g × K (for both heating/cooling), for RT24, RT25, RT26 and PureTemp 25. In the latent responses, pronounced peaks developed that represent the solid–liquid and liquid–solid phase changes of the various PCMs.

**Figure 12:**
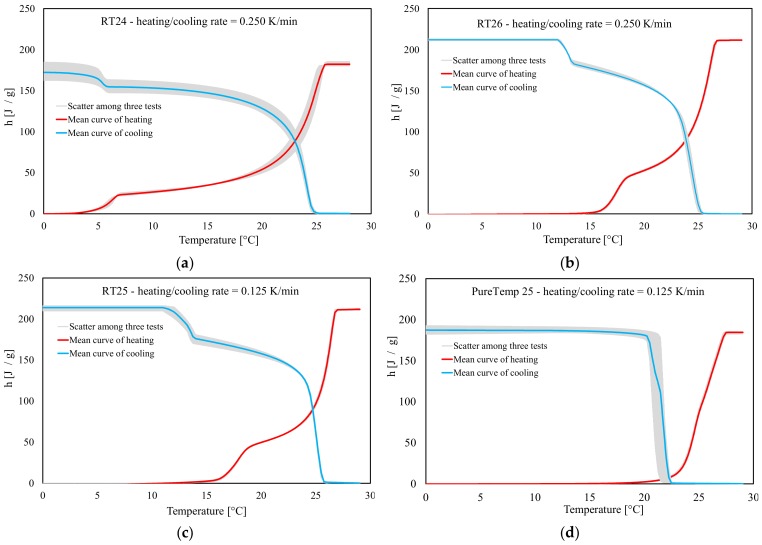
Enthalpy (latent-only) measurements (heating and cooling) following the dynamic DSC tests: (**a**) RT24, (**b**) RT26, (**c**) RT25 and (**d**) PureTemp 25.

**Figure 13:**
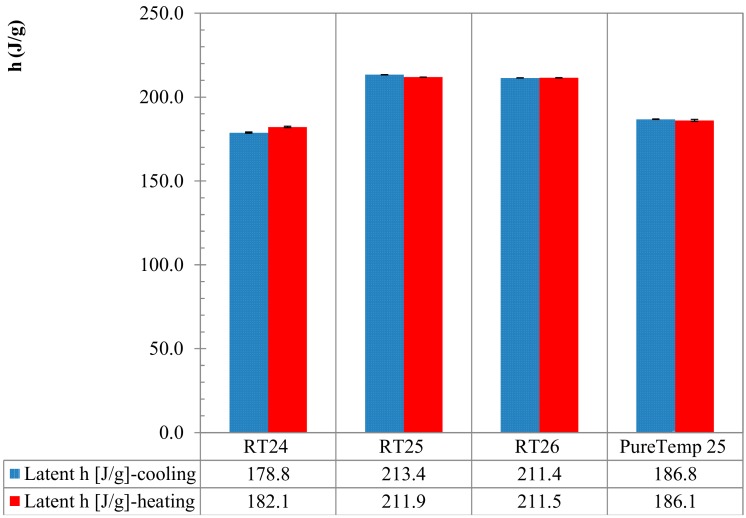
Latent absorbed/released h [J/g] during melting/crystallization following the dynamic DSC method.

In Figure 12, the resulting enthalpy curves (latent part only), measured with the dynamic DSC method, are shown for all materials. The plotted curves represent the mean values of the measurements done for three samples, over three cycles. These data mainly represent the absorbed/released thermal energies for heating and cooling, respectively, and the unitary latent heat during a phase change. The measured total and specific *h* of each material is shown in Figure 13. It can be observed that the absorbed heat from 0 J/g to the total specific latent heat of each PCM (i.e., 182.1 J/g for RT24, 193.3 J/g for RT25, 211.9 J/g for RT26 and 186.1 J/g for PureTemp 25) is represented by a clear non-isothermal behavior. Similar trends, in a releasing way, from the maximum values of *h(T)* (i.e., form 178.8 J/g for RT24, 213.4 J/g for RT25, 211.4 J/g for RT26 and 186.8 J/g for PureTemp 25) to 0 J/g, can be observed for the cooling responses. From the data, it can be observed that the bio-based PureTemp 25, compared to the RT ones, is characterized by a faster acceleration of the *h(T)* response. This means that the phase change occurs in a narrower melting or crystallization range, which is often more appreciated in practical passive applications for buildings.

**Figure 14:**
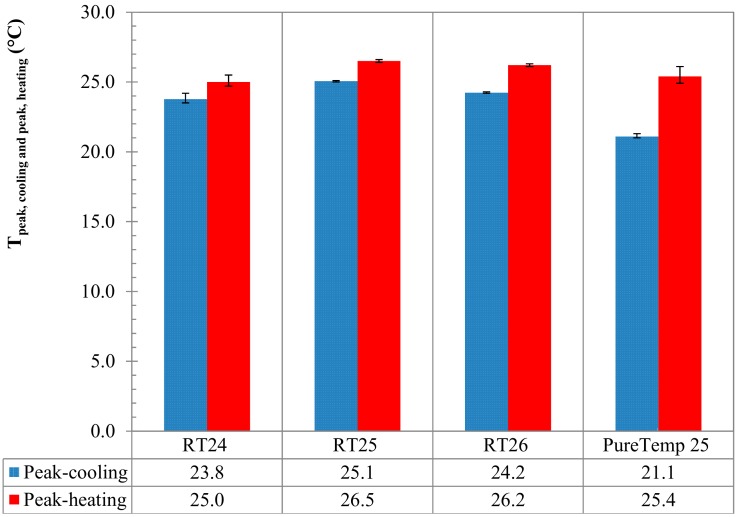
Comparison of the maximum peak temperatures (°C) of all samples in the melting/crystallization range.

Finally, the maximum peak temperatures (meaning those points at which the maximum *c_p_* was registered) for either melting or crystallization of all PCMs are compared in Figure 14. The graphs further show the “melting” and “crystallization” temperatures of the three specimens, their mean values and the statistical deviation via error bars. It can be seen that the peak temperature (mean value of three specimens) at which melting happened were detected at 25.0, 26.5, 26.2 and 25.4 °C for RT24, RT25, RT26 and PureTemp 25, respectively. Similarly for the crystallization measurements (again mean value of three specimens), peak temperatures were registered at 23.8, 25.1, 24.2 and 21.1 °C for RT24, RT25, RT26 and PureTemp 25, respectively.

In Figure 14 it can also be seen that there is an obvious difference between the cooling and heating peak temperature. Overheating or subcooling effects [18], occurred due to a kinetic delay in phase transformation, could be a possible explanation for the shifted heating/cooling peaks. Upon the attained results, the subcooling is lower in paraffins, also known as alkanes (about 1.2–2 K), compared to the PureTemp 25 (made of fatty acids) with about 4.3 K subcooling. These phenomena occur even if the amount of stored/released heat stayed almost the same (see Figure 13).

Finally, the T_onset,heating_ and T_onset,cooling_ are reported in Table 3. They are representing the extrapolated peak start temperature (defined *T_p,ini,e_* as sample transformation temperature by DIN 51007:2019 [39]). These temperature values represent the intersection point of the extrapolated start baseline with the tangent (or a straight) line through the linearly rising or falling part of the endothermic or exothermic peak. The onset temperatures are worth knowing since at this point the lower surface area of the sample (crucible bottom) begins to change phase, as this temperature can be determined sufficiently reproducible and is almost independent of the heating rate. All other temperatures largely depend on test conditions, masses and heating/cooling rates (as shown in Figure 5, Figure 6, Figure 7 and Figure 8).

## 4. Stepwise DSC Results

### 4.1. Heating Rate Effect

Tests with different heating rates (ranging from 2 K/min to 0.125 K/min) have also been performed for the stepwise DSC method. These activities were scheduled for RT25 and PureTemp 25 under temperatures ranging between 10 and 30 °C (a range which is relevant for the phase transition).

**Figure 15:**
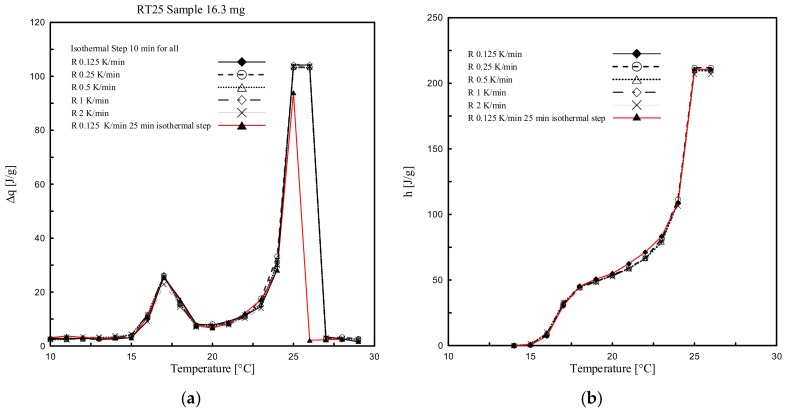
(**a**) ∆*q (T)* and (**b**) *h(T)* curves determined through stepwise DSC and considering different heating rates for RT25.

**Figure 16:**
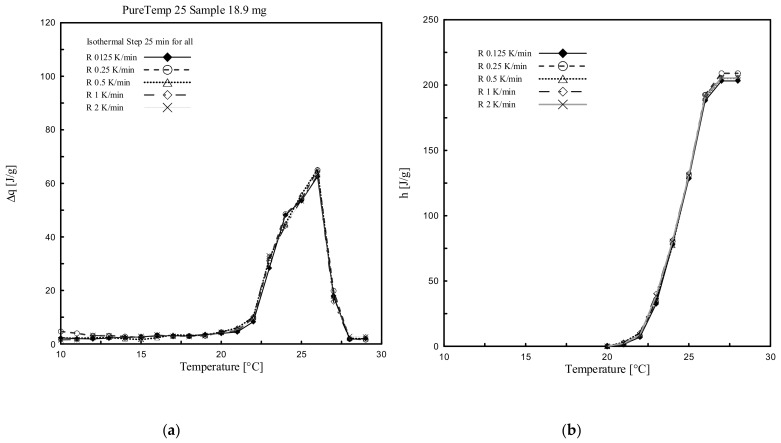
(**a**) ∆*q (T)* and (**b**) *h(T)* curves determined through stepwise DSC and considering different heating rates for RT25.

Figure 15a and a show the stored heat, per each temperature interval (being 1 K, representing the difference between two isothermal temperature increments), while the accumulated specific enthalpy (latent part only) is shown in Figure 15b and Figure 16b. Actually, these results represent the determined partial enthalpies and the cumulated ones between 10 °C and 30 °C from the measurements done for RT25 and PureTemp 25.

It is shown in Figure 15 and Figure 16 that all the results, having different heating rates, are completely overlapping. Particularly, the ∆*q-T* and *h-T* (dotted) curves obtained by considering different heating rates, i.e. 2, 1, 0.5, 0.25 and 0.125 K/min, show almost the same values, with a very small difference in terms of either ∆*q or h* values. The enthalpy deviation between the different heating curves has a maximum of 5 J/g, and this occurred mainly close to the melting range. This difference is less than 3% of the total and specific heat absorbed in the considered temperature range. This means that with the stepwise method the influence of the heating rate, which classically affects dynamic DSC measurements, can be neglected. (see Section 3.1 where the results were obtained through dynamic DSC measurements).

It may be worth mentioning that the length of the relaxation time (namely, isothermal step) is an important parameter for the temperature resolution of the accumulated heat, especially in the temperature ranges close to the melting peak (defined as the point in which the maximum amount of stored heat can be measured). Particularly, to reach thermal equilibrium in these zones a longer isothermal step is required. This can be observed in Figure 15a where the highest peak (readable in the ranges 25 °C and 26 °C) for all tests is obtained for heating rates of 2, 1, 0.5, 0.25 and 0.125 K/min, while performed with an isothermal step of 10 min. To evaluate the sensitivity of the results regarding the duration of the applied isothermal step, an additional measurement was conducted for a heating rate of 0.125 K/min and where the isothermal step was increased from 10 min to 25 min with the aim to reach proper thermodynamic equilibrium during the phase change transition (see the red curve in Figure 15). The results clearly show the influence of this increased isothermal step.

### 4.2. Enthalpy and Phase Change Temperature

The enthalpy measurements, i.e., the absorbed specific heat along the considered temperature steps, and phase change temperatures obtained by an adopting isothermal step time of 25 min and various heating rates, are reported in this subsection. Both sensible and latent absorbed heat, in the range of 10 °C and 30 °C, are evaluated.

**Figure 17:**
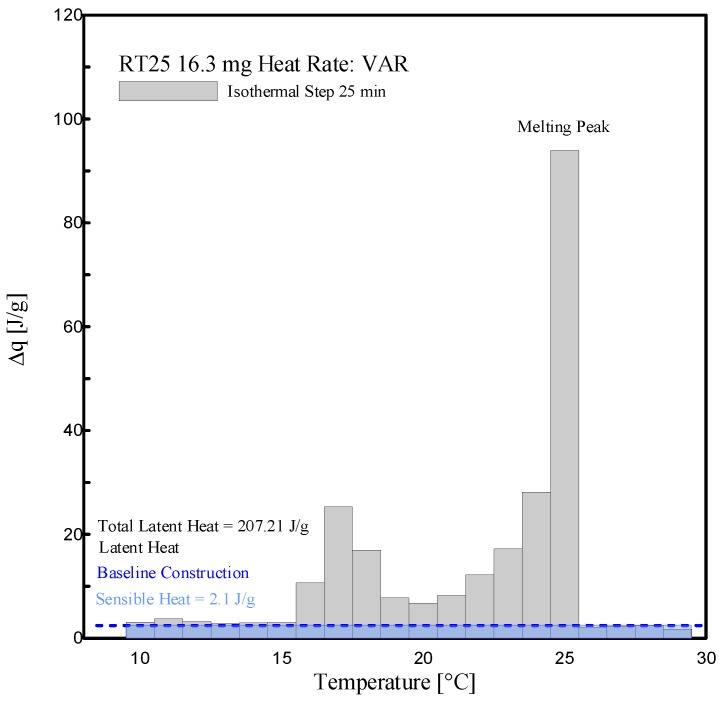
Histogram view of stored heat ∆*q (T)* for RT25, at several temperature intervals and adopting an isothermal time step of 25 min.

**Figure 18:**
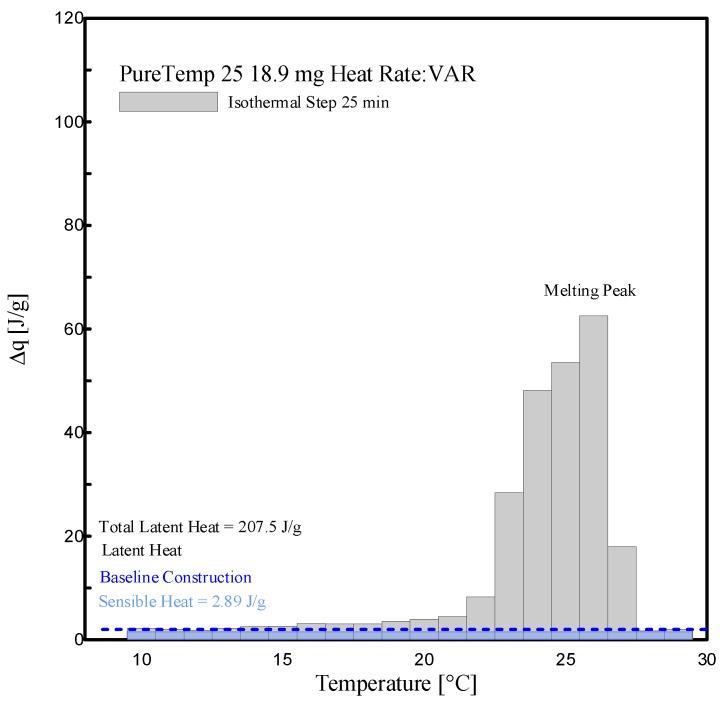
Histogram view of stored heat ∆*q (T)* for PureTemp 25, at several temperature intervals and adopting an isothermal time step of 25 min.

Figure 17 and Figure 18 show a histogram of the stored heat, ∆*q (T)*, between the temperature intervals, representing the temperature resolution of the acquired data, of 1 K, for the aforementioned tests. The corresponding tabular data is provided in Table 4. From the results, it can be seen that the melting of RT25 mainly starts at 15 °C and ceases at 26 °C. A baseline construction was used to evaluate and separate the sensible part form the latent one. The sensible absorbed heat was evaluated at 2.10 J/g, which was in agreement with the value declared in the datasheet, i.e., 2 J/g, (see Table 1). The melting peak temperature as indicated in Figure 17 can be appreciated in the range of 25–26 °C. The (latent) melting of the PureTemp 25 takes place in a smaller temperature interval (from 21 °C to 28 °C, see Figure 18). The absorbed sensible heat of PureTemp 25 was evaluated to 2.89 J/g while its melting point falls in the range of 25–26 °C.

The phase transition enthalpy (considering the latent part only), in the defined melting range of 15 °C to 26 °C and constructing the baseline at 15 °C, was 207.21 J/g for RT25 (see Table 4). This value is slightly different to the one measured through the dynamic DSC measurements (211.9 J/g reported in Figure 13). The total latent heat of PureTemp 25, evaluated in the temperature range between 21 °C and 28 °C, is 207.5 J/g (see Table 4), which somehow deviates from the value measured using the dynamic DSC method (186.1 J/g, showed in the Figure 13). However, it may be worth mentioning that the results obtained from dynamic DSC tests or the stepwise methods are highly depending on the baseline construction, which separates the sensible heat from the latent part. As the baseline-construction can be done more precisely for the dynamic DSC method rather than for the stepwise one, the final results of the latent heat by using both the aforementioned methods might be significantly affected.

From the results it can be stated that the stepwise DSC method is capable of cleaning and eliminating the heating rate effects, which is classically of interest for dynamic DSC measurements. It is a more appropriate method that is able to capture the total enthalpy in the defined temperature range by applying predefined temperature steps, and also for validating the melting peak (range) more precisely than with dynamic DSC tests. However, some drawbacks are still characterizing the stepwise DSC method, which is mainly that the temperature resolution adopted in these approaches is still too high. The smaller the temperature steps, the better the temperature resolution. However, the measuring time could be considerably longer as the step sizes decrease. In general, the shape characterization of *c_p_(T)* curves can be achieved faster (and more properly) by using the dynamic DSC method than the stepwise one.

In summary, it can be observed that the temperature resolution for DSC tests depends on the amount of analyzed intervals, needed to measure the heat storage capacity, within the considered temperature range. Based on the chosen DSC method the “resolution” of the data, representing the heat supplied to the sample and the corresponding temperature range, can differ. In the stepwise method heating is applied in small intervals called “steps”. Thus, the resolution is equal to the height (or length) of the temperature steps, however it is required that the start and end of each step must be in an isothermal state. For dynamic DSC tests, the interval is continuously scanned by heating of the sample. There are actually no steps waiting for thermodynamic equilibrium. This means that the heating rate needs to be low enough to assure thermodynamic equilibrium within the entire sample. Theoretically, this will occur by employing a heating rate close to 0 K/min., which is practically not feasible. This means that the heat supplied to the sample, according to each data recording (equal to the enthalpy change), cannot be fully assigned to the measured temperature. Too high heating rates may cause significant errors (less “accuracy” of the results) in the data of the heat stored as a function of temperature. However, in DSC dynamic tests the resolution of the data itself is only restricted by the data recording system.

## 5. Conclusions

In this work, a detailed experimental program is reported for analyzing the Thermal Energy Storage (TES) capacity of three paraffin-based and one bio-based PCM, employable for construction and buildings applications. For this aim, the paraffin-based waxes, RT24, RT25 and RT26, and an eco-friendly bio-based PCM, PureTemp 25 were examined using DSC testing equipment. All selected PCMs have melting/ crystallization temperatures within the well-known comfort zone temperature for buildings, e.g., ranging between 19 and 26 °C. Heat storage capacities, melting/ crystallization responses and enthalpies, under both sensible (solid and liquid) and latent TES responses, were investigated through DSC tests.

Two alternative methods were performed and compared, i.e., a dynamic DSC and a stepwise DSC method. Based on the results reported in this work, the following conclusions can be drawn:

- Dynamic DSC tests, performed for all 4 different PCMs and considering several heating rates (ranging from 10 K/min to 0.125 K/min), showed that the measurable melting peak temperatures *T_m_* (indicated as that point at which the maximum peak of *c_p_(T)*, or alternatively the inflection point of *h(T),* can be registered), mainly depends on the heating rate. *T_m_* can be shifted progressively to lower values as the heating rate decreases and vice versa;
- The *c_p_(T)* and *h(T)* curves under dynamic DSC strongly depend on the selected heating rates. Only the related measure of the accumulated (total) specific (unitary) latent heat is independent to the heating rate. In general terms, the total achievable *h* is the same when different heating rates are assumed, even though the *h(T)* curves and their slopes, *cp(T)*, are heating rate dependent;
- Dynamic DSC tests, performed by considering 2 different (low and high) sample masses, for RT25 and PureTemp 25, highlighted that the specimens with a lower mass are less affected by the heating rate. However, they suffer higher measurement noise and less representativity of the material under investigation;
- Dynamic DSC results of higher mass samples showed more stable measurements, especially in the sensible parts, and seemed to be more representative for the behavior of the investigated PCMs. However, they turned out to be more sensible to heating rate effects under latent heat storage.
- It can be concluded that the selection of the proper “heating rate” and “sample mass” (aimed at fulfilling the IEA requirements) arises from a compromise between accuracy of the measurements (i.e., enthalpy, specific heat capacities and melting point) and the mitigation of the heating/cooling rate effects.
- It can be stated that reliable and reproducible results can be achieved for characterizing the aforementioned (paraffin- and bio-based) PCMs by following the IEA standard procedure and adopting the dynamic DSC method.
- Results following the stepwise method demonstrated that both ∆*q(T)* and *h(T)* results, obtained by considering several heating rates (2, 1, 0.5, 0.25 and 0.125 K/min), presented almost the same trend and similar values (with differences significantly less than 0.2 °C and/or in terms of enthalpy of max. 5 J/g between them). This means that with the stepwise method the heating rate influence, which classically affects the dynamic DSC, can be fully controlled.
- The main drawbacks of the stepwise method, however, are related to its enormously time-consuming character, imposed by the test procedure. The required length of the relaxation time (namely, isothermal step) is sometimes too high to achieve proper results, especially in the melting region.
- The temperature resolution of the stored heat is considerably higher, using the stepwise method, in comparison to the dynamic one. However, a maximum resolution of 1 K, as those employed in the stepwise method of this study, is not high enough to obtain the *c_p_(T)* curve of the material, which would require smaller discretized temperature steps, and also does not allow describing the melting temperature of the material in a concise way.
- The results obtained following both dynamic and stepwise methods are dependent on the baseline construction, which allows separating the sensible from the latent heat part. In this regard, the baseline-construction can be more precisely built in the dynamic method than the stepwise one, because more valuable measurement data can be evaluated within very small temperature steps. For this reason, the stepwise method is more appropriate to measure the total enthalpy (both sensible and latent heat) in defined temperature steps and also validating the melting peak reached from the dynamic procedure, while the dynamic measurements can ascertain the stored latent heat much more quickly and precisely. Then using the IEA Standard, dynamic measurements are less time-consuming and also more precise to characterize the melting behavior of the material.

Further experimental characterizations of the TES capacity of several other types of bio-based PCM, to be employed as environmentally friendly substitute of petroleum-based PCM in cement-based systems, are currently under development.

## Figures and Tables

**Table 1:** Properties of RT24, 25, 26 and PureTemp25 as given by manufacturers´ datasheets [36,37].

Properties	RT24	RT25	RT26	PureTemp25
*T_m_* (main peak) [°C]	24	25	26	25
Density liquid [kg/L]	0.77 (40 °C)	0.76 (at 40 °C)	0.75 (at 30 °C)	0.86
Density solid [kg/L]	0.88 (15 °C)	0.88 (at 15 °C)	0.90 (at 20 °C)	0.95
Specific Heat Capacity (liquid) [kJ/kg×K]	-	-	-	2.29
Specific Heat Capacity (solid) [kJ/kg×K]	-	-	-	1.99
Spec. Heat Capacity [kJ/kg×K]	2	2	2	-
Heat storage capacity [kJ/kg]	160 (16–31 °C)	170 (16–31 °C)	180 (19–34 °C)	187 *

*Measurement interval not available in the Datasheet.

**Table 2:** Samples, heating rates, investigated parameters and DSC test-type applied in this study.

Tests	Materials (Mass Values in mg)															
	RT24		RT25				RT26				PureTemp25					
**Dynamic Heat Rate (R), (R: 10, 5, 2, 1, 0.5, 0.25, 0.125 K/min)**	P1	P3	P3	P1	P2	P3		P4	P1	P2		P3	P1	P2	P3	P4
	✔	-	-	✔	-	-		-	✔	-		-	✔	-	-	-
	18.8			17.2					17.7				18.9			
**Dynamic Sample Mass** **(R: 0.125, 0.25 K/min)**	-	-	-	✔	✔	✔		✔	-	-		-	✔	✔	✔	✔
				16.3	18.4	18		7.9					18.9	19	17.7	10.1
**Dynamic - Enthalpy Temperature h (T)** **(R: 0.125 K/min)**	✔	✔	✔	✔	✔	✔		-	✔	✔		✔	✔	✔	✔	-
	13.3	16.5	16.9	16.3	18.4	18			17.7	18.5		16.1	18.9	19	17.7	
**Dynamic - Heat Capacity c_p_ (T) (R: 10 K/min)**	✔	✔	✔	✔	✔	✔		-	✔	✔		✔	✔	✔	✔	-
	14.3	16	16.1	17.2	18.4	18			16.4	18.9		12.4	10.5	18.9	18	
**Step-wise Heat Rate (R:2, 1, 0.5, 0.25, 0.125 K/min)**	-	-	-	✔ 16.3	-	-		-	-	-		-	✔ 18.9	-	-	-
**Step-wise - Enthalpy Temperature h (T) (R: VAR)**	-	-	-	✔ 16.3	-	-		-	-	-		-	✔ 18.9	-	-	-

Abbreviations in table: R: heating Rate; h: enthalpy; c_p:_ specific heat capacity; VAR: variable; RT: RubiTherm.

**Table 3:** Comparison of extrapolated peak initial temperatures (assigned to the transformation temperature) of all samples in the melting/crystallization range according to DIN 51007 [39].

PCM Type	RT24 R 0.250 K/min		RT25 0.125 K/min		RT26 0.250 K/min		PureTemp 25 0.125 K/min	
	T_onset, heating_	T_onset,_ _cooling_	T_onset, heating_	T_onset, heating_	T_onset, heating_	T_onset, heating_	T_onset, heating_	T_onset, heating_
**P_1,2,3_**	3.0	24.3	15.5	25.7	15.6	25.1	23.0	21.9

**Table 4:** Stored heat values (sensible = S, latent = L and total = S + L) of RT 25 and PureTemp 25, as shown in Figure 17 and Figure 18.

Temp. Range [°C]	RT25 (Mass: 16.3 mg)		PureTemp25 (Mass: 18.9 mg)	
	∆q (total: S + L)	∆q (L)	∆q (total: S + L)	∆q (L)
	[J/g]	[J/g]	[J/g]	[J/g]
15 to 16	3.03	0.93	-	-
16 to 17	10.70	8.60	-	-
17 to 18	25.33	23.23	-	-
18 to 19	16.96	14.86	-	-
19 to 20	7.77	5.67	-	-
20 to 21	6.77	4.67	-	-
21 to 22	8.26	6.16	4.48	1.59
22 to 23	12.18	10.08	8.30	5.41
23 to 24	17.23	15.13	28.39	25.50
24 to 25	28.13	26.03	48.16	45.27
25 to 26	93.95	91.85	53.53	50.64
26 to 27	-	-	62.56	59.67
27 to 28	-	-	22.32	19.43
-	230.31	**207.21**	227.74	207.48
